# [^18^F]FDG-PET/CT in patients with bacteremia: Clinical impact on patient management and outcome

**DOI:** 10.3389/fmed.2023.1157692

**Published:** 2023-03-29

**Authors:** Søren Hess

**Affiliations:** ^1^Department of Radiology and Nuclear Medicine, Esbjerg Hospital – University Hospital of Southern Denmark, Esbjerg, Denmark; ^2^Department of Regional Health Research, Faculty of Health Sciences, University of Southern Denmark, Odense, Denmark; ^3^IRIS – Imaging Research Initiative Southwest, Esbjerg, Denmark

**Keywords:** [^18^F]FDG-PET/CT, infection, bacteremia, blood stream infection (BSI), PET

## Abstract

Bacteremia is the presence of viable bacteria in the bloodstream, a complicated and potentially dangerous systemic medical condition that may range from asymptomatic and clinically relatively indolent cases to more severe bloodstream infection (BSI) and ultimately life-threatening septic shock with fatal outcome. BSI is classified as simple (bacteremia only) or complex (BSI with metastatic spread), and the morbidity is higher in the latter, probably due to insufficient eradication. Treatment of simple BSI is usually short-term antibiotic courses, whereas complex BSI with metastatic foci requires more advanced treatment including long-term antibiotics or invasive drainage to gain infection control. Thus, identifying metastatic infection has an important clinical impact but remains a challenge; only half of the patients progress to complex BSI, and many patients present without relevant signs or symptoms, so imaging is pivotal. This review summarizes the potential role and recommendations of [^18^F]FDG-PET/CT in BSI, based on the relatively sparse and heterogeneous literature. [^18^F]FDG-PET/CT should be considered in suspected complex BSI, in patients at high risk of metastatic spread, and in BSI in ICU patients. [^18^F]FDG-PET/CT has an impact on patient management, treatment strategy, and patient outcome, mainly by directing the diagnostic process toward more specific diagnostics or by modifying treatment regimens resulting in reduced relapse rates and reduced mortality. Finally, a negative scan may obviate the need for further workup.

## Introduction

Bacteremia or bloodstream infections (BSI) are common and difficult clinical entities with potentially serious consequences including death. Some reports state that BSI occurs in 5–10% of hospitalized patients with overall mortality higher than 15% (1, 2).

In fever of unknown origin, the key issue is usually to establish the focal point of the fever. In BSI, another and perhaps clinically more important issue is to distinguish between simple bacteremia (BSI only) and complex (or metastatic) bacteremia (BSI with solid or metastatic foci). A significant proportion of patients progress from simple bacteremia to complex bacteremia; studies on *Staphylococcus aureus* report metastatic infection in 16–73% of patients (3, 4). Complex bacteremia requires a different treatment strategy, i.e., escalation (added/combined drug regimens), elongation (prolonged treatment for 4–6 weeks depending on the location of foci), and/or surgical interventions (prosthesis removal or drainage of deep tissue abscesses). Correspondingly, insufficient eradication leads to relapse in approximately 15% and an increase in mortality, but in one-third of cases, the metastatic foci remain asymptomatic (5). Thus, a great deal of work is going into differentiating patients at risk of complex BSI — if they are identified, guidelines usually suggest prolonged treatment to prevent or reduce the risk of fulminant bacterial spread. Risk factors for developing complex BSI include community acquisition, prolonged or persistent symptoms/findings, and prosthetic implants (3). However, 35–50% of all patients at high risk of complex BSI never progress to spread, and as such, as many as 50% of patients are over-treated to prevent spread (4).

Bacteremia is divided into gram-positive and gram-negative bacteremia; gram-positive bacteremia is more common than gram-negative ones. This may be attributable to the increased use of prosthetic devices, intravenous catheters, invasive procedures, and the widespread use of antibiotic prophylaxis with fluoroquinolones that all predispose to gram-positive growth (6).

In gram-positive bacteremia, there are some characteristic associations between species and sites of focal infections, e.g., pneumococci and pneumonia, whereas *S. aureus* infection sites are often more occult, and therefore more commonly encountered as the culprit species in BSI of unknown origin. Gram-negative bacteremia is often caused by *Escherichia coli* and other *Enterobacteriaceae*. It is usually associated with gastrointestinal or urinary tract infections, whereas gram-negative species have much less ability to adhere to prosthetic material than gram-positive ones (6, 7). These pointers should be kept in mind when assessing imaging, but most species can give rise to BSI anywhere, so one must keep an open mind.

Further division is based on where the patient most likely contracted the infectious microorganism: community-acquired BSI (CA-BSI) is usually defined as outpatients or patients with confirmed BSI <48 h post-admission, whereas nosocomial BSI (N-BSI) is usually defined as confirmed BSI >48 h post-admission. In CA-BSI, gram-negative is more prevalent, whereas gram-positive is more prevalent in N-BSI (1, 8).

### Some specific scenarios

*Staphylococcus aureus* is probably the most commonly encountered BSI by the nuclear medicine physician in referrals to [^18^F]FDG-PET/CT. First, the incidence of *S. aureus* BSI is generally increasing due to an aging population with heart valve prostheses and joint prostheses that are common predilection sites. Second, *S. aureus* has an inherent ability for metastatic spread and progression to complex BSI that require more advanced treatment to reduce the risk of recurrence, prolonged disease, or mortality that may reach 20–40% (9, 10). *Staphylococcus aureus* BSI comprises 20% of N-BSI, and metastatic spread is a dreaded complication (9). Most common are infectious endocarditis, osteomyelitis and joint infections (including prosthetics), and deep tissue abscesses — all trigger treatment modification (i.e., escalation or elongation of antibiotic regimens or surgical interventions) (11). A special entity is *S. aureus*-associated pneumonia, which accounts for 8% of all BSI, 30% of N-BSI, and 68% of all BSI in the intensive care unit (ICU). Overall, BSI is established in 5–15% of community-acquired pneumonia and 24–36% of ventilator-associated pneumonia, whereas the rate of BSI is approximately 60% in both community-acquired *S. aureus* pneumonia and nosocomial *S. aureus* pneumonia in the ICU. In the former, BSI usually occurs early in the course, whereas in the latter case, pneumonia may be ventilator-associated or secondary to influenza, and therefore, BSI may appear rather late (11). Thus, it is clinically relevant to differentiate between bacteremia secondary to *S. aureus* pneumonia and pneumonia secondary to *S. aureus* BSI, so even with well-known pneumonia in ICU patients, an [^18^F]FDG-PET/CT late in the course may contribute to localizing metastatic foci including the lungs.

Regarding the ICU, the incidence of BSI is at least double that of the general hospital population and mortality is at least 3-fold and described as the leading cause of death in the ICU. Patients are evaluated for foci with the aim of source control, e.g., drainage, but despite extensive workup, foci often remain occult. [^18^F]FDG-PET/CT is difficult in the ICU setting due to multiple interfering factors leading to reduced scan quality, e.g., multiple intravenous drugs (some dissolved in glucose), generally difficult blood glucose control, and frequently poor kidney and/or liver function (2, 12). The practical setup is also challenging. At our institution, we usually inject FDG in the ICU, and patients are only in the PET center for the scan, but even then, [^18^F]FDG-PET/CT takes significantly longer than a CT, which puts a strain on ICU resources. Finally, ICU patients are usually hooked up to monitors, ventilators, injection pumps, etc., which may hamper imaging or even the possibility to pass through the scanner. [^18^F]FDG-PET/CT scans in ICU patients need planning and close cooperation between the specialties.

Febrile neutropenia is a dreaded complication in oncology and hematology, but only 30–50% is due to infection, and BSI is only present in 25–30%. In many cases of febrile neutropenia, fever is caused by chemotherapy or tumor fever, and there is no effect from antibiotics. On the other hand, in approximately 50% of febrile neutropenia patients with BSI, the bacteremia is complex with focal spread, and prolonged treatment is warranted just as in non-oncologic BSI (13).

### Aim

This review explores the potential impact of [^18^F]FDG-PET/CT on subsequent treatment strategies in both simple and complex BSI. A positive PET/CT scan may direct the diagnostic process toward more specific diagnostics. A more direct impact on management is the modification of treatment, for instance, as a guide for invasive treatment, e.g., abscess drainage. It may also cause escalation or elongation of therapy based on positive findings or even de-escalation following negative scans. Sometimes a negative scan may also prompt a switch from intravenous to oral regimens in limited disease. Thus, [^18^F]FDG-PET/CT may directly influence patient prognosis through its impact on treatment modifications if better focal control reduces morbidity, relapse rates, and mortality (Table 1).

**Table 1:** Overview of studies evaluating [^18^F]FDG-PET/CT in bloodstream infections.

Study	Population	*N*	Study design	Major findings
**Studies evaluating the impact of [**^**18**^**F]FDG-PET/CT on treatment**				
Brøndserud et al. (14)	BSI with Gram-positive cocci or SA	157	Retrospective	Change in therapy in 15%
van Leerdam et al. (15)	High risk SA-BSI	132	Retrospective	Change in therapy in 17%
Tsai et al. (16)	BSI with mixed species	102	Retrospective	Change in management in 45%
Pijl et al. (2)	BSI in ICU-patients	30	Retrospective	Change in management in 47%
Kluge et al. (12)	BSI in ICU-patients	18	Retrospective	Change in management in 33%
**Studies evaluating the impact of [**^**18**^**F]FDG-PET/CT on patient outcome**				
Vos et al. (5)	BSI at risk of metastatic spread	115	Prospective	PET increased detection of metastatic foci, reduced relapse rate, and reduced mortality
Berrevoets et al. (4)	High risk BSI with negative PET and echocardiography vs. simple BSI	76	Retrospective	Comparable relapse rates and mortality in simple BSI vs. high risk BSI
Yildiz et al. (17)	High risk BSI with or without PET	102	Retrospective	PET resulted in reduced mortality compared to controls
**Studies evaluating the impact of [**^**18**^**F]FDG-PET/CT on management and patient outcome**				
Ghanem-Zoubi et al. (18)	SA-BSI with/without PET	149 × 2	Prospective	PET resulted in longer courses of antibiotics and better mortality compared to controls
Berrevoets et al. (3)	High risk BSI	99	Retrospective	PET changed treatment in 73% and reduced mortality
Kouijzer et al. (19)	Complicated BSI with negative PET and echocardiography	106	Retrospective	Switch from intravenous to oral antibiotics is safe and reduce duration of hospital stay

BSI, bloodstream infections; SA, *Staphylococcus aureus*; SA-BSI, *Staphylococcus aureus* bloodstream infections; PET, [^18^F]FDG-PET/CT.

## Impact on patient management

Several studies have looked at the overall impact of [^18^F]FDG-PET/CT on treatment modifications. Brøndserud et al. (14) performed a retrospective study of consecutive patients with confirmed BSI with *S. aureus* and gram-positive cocci other than pneumococci (as they usually present themselves fairly easily). All patients underwent an [^18^F]FDG-PET/CT scan in the search for infectious foci, and besides the overall diagnostic yield of [^18^F]FDG-PET/CT, they also evaluated any relevant clinical impact defined as cases where PET results identified foci as the first modality or when findings led to treatment modifications. They found metastatic foci in 56% and high clinical impact in 47% of the 157 included patients, including changes in therapy in 15%. When patients experience long disease courses, some fear a potential negative impact from antibiotic treatment on [^18^F]FDG-PET/CT efficacy, but in this study, the duration of antibiotic treatment neither influence the clinical impact nor the time interval from confirmed BSI to [^18^F]FDG-PET/CT.

Patients are often subjected to a wide range of futile examinations prior to PET. Reducing these by performing [^18^F]FDG-PET/CT earlier would benefit the individual patient and health economics. In this study, patients underwent a median of four futile investigations prior to PET (with a range of 0–12), albeit the study was not specifically designed to evaluate this particular issue.

The most recent study by van Leerdam et al. (15) found similar results with regard to additional foci and change in treatment in a retrospective cohort of patients with *S. aureus* BSI suspected to be complex and therefore pre-planned antibiotic treatment of >6 weeks. They included 132 patients who underwent [^18^F]FDG-PET/CT and found additional metastatic foci in 52%. The original treatment plan changed in 17%, primarily due to the confirmation or rejection of an infected vascular graft or thrombus. The treatment regimen was shortened in two cases where suspected vascular thrombus infection and arthritis were ruled out. Antibiotic treatment was switched from an intravenous to an oral regimen in three cases with ruled-out infected thrombus but confirmed pulmonary metastatic foci. In 13 cases, intravenous treatment was extended (and in four of these additional rifampicin was also added) due to confirmed infection in vascular or joint prostheses, endocarditis, osteomyelitis, and/or soft tissue abscess. Thus, [^18^F]FDG-PET/CT could individualize and refine the treatment of *S. aureus* BSI planned for generic long-term antibiotic regimens.

In another retrospective, single-center study, Tsai et al. (16) included 102 patients BSI who underwent [^18^F]FDG-PET/CT within 1 week of diagnosis. In total, 73% of the scans were positive; the most common organs/tissues were vertebral osteomyelitis/spondylodiscitis (21%) and lung (21%). They found a 3-fold higher impact on management compared with the previous study, i.e., 45% (40 PET+/6 PET−), but in most cases, the modified management comprised referral for subsequent imaging. A low number of PET-negative patients were referred for subsequent imaging, which is an important point; a negative [^18^F]FDG-PET/CT may reduce the need for further imaging, as also suggested by the aforementioned results from Brøndserud et al.

To avoid over-utilization of PET scans, it is important to stratify which patients to refer for [^18^F]FDG-PET/CT. Tsai et al. found increased CRP associated with positive scans, but the best cutoff of 54 mg/ml yielded a sensitivity of only 79% with a specificity of 65%.

Pijl et al. (2) included a retrospective, consecutive cohort comprising all ICU patients with proven gram-positive BSI or gram-negative BSI and an [^18^F]FDG-PET/CT. They included 30 patients with a median CRP of 114 mg/L. *S. aureus* was the predominant cause (37%) and mortality was 30%. Many patients underwent examinations prior to PET, e.g., X-ray (100%), ultrasonography (93%), CT (71%), and MRI (17%). As a special feature, PET scans were quality assessed based on background, motion artifacts, suppression of physiologic uptake, and overall readability; 70% was deemed adequate and 30% was of poor quality.

In the 21 PET-positive scans, the most common findings were pneumonia or arthritis. Overall, [^18^F]FDG-PET/CT led directly to a change of management in 14 out of 30 patients (47%). On regression analysis, only PET quality was associated with positive findings (reduced image quality led to fewer PET positives), whereas CRP, duration of antibiotics, duration of admittance to ICU, or mechanical ventilation did not impact diagnostic yield. This is in keeping with some of the findings mentioned previously.

This study also points to the potential of employing PET earlier in the course to limit futile tests.

Finally, [^18^F]FDG-PET/CT impacted test probability. Overall pretest probability for infection was 73%, whereas the post-test probability was 95% (in PET+) and 22% (in PET−), respectively.

These results were corroborated by an older study by Kluge et al. (12). They retrospectively included 18 ICU patients with severe septicemia without known focus. In total, 12 had BSI, and all were extensively worked up prior to [^18^F]FDG-PET/CT. The median time from ICU admission to [^18^F]FDG-PET/CT was 11 days, and 17 out of 18 patients received antibiotics at the time of PET.

The PET results led to therapeutic changes in six patients (33%), i.e., surgery in two, pacemaker removal in two, and initiation or prolonged antibiotics in two cases.

## Impact on patient outcome

Another approach is the impact on patient outcomes. This was the focus of one of the first large studies on [^18^F]FDG-PET/CT in bacteremia by Vos et al., probably the first prospective one (5). It was a case–control study of patients with gram-positive BSI, with risk factors of metastatic spread and with or without a PET scan. Case patients received PET-directed treatment: no foci prompted 14-day treatment, whereas foci prompted prolonged treatment for 6–12 weeks depending on the location of the foci. They compared 115 cases with 230 controls.

Overall, they found metastatic foci in 68% of cases vs. 36% of controls, especially endovascular, spinal, and pulmonary lesions. These foci not only require prolonged antibiotic regimen or invasive intervention, but they also only rarely have localizing symptoms. Overall, the relapse rate was 2.6% in cases versus 7.4% in controls — this was not statistically significant, although the study was designed to detect a 10% decrease, in a subgroup of *S. aureus*-related relapses, the difference reached statistical significance. All-cause 6 months mortality was lower in cases (19%) than in controls (32%).

The two groups were comparable according to baseline characteristics, but two factors stood out: treatment delay was more prevalent in controls (45% vs. 27%), although it did not influence mortality when added as a covariate. The second potential confounder was a higher prevalence of prolonged BSI in the intervention group, which could skew patients toward a higher relapse rate, but they did not obtain continuous blood cultures routinely in the controls, so the true effect of this is equivocal.

Berrevoets et al. (20) looked at the value of a negative [^18^F]FDG-PET/CT in a retrospective case–control setting. They compared patients with *S. aureus* BSI and risk factors for complex BSI but negative echocardiography and negative [^18^F]FDG-PET/CT (36 cases) vs. patients with simple BSI (40 controls).

As mentioned earlier, the same prolonged treatment is recommended for manifest complex BSI and patients with risk factors for developing complex BSI. Moreover, as also mentioned previously, 35–50% of patients with risk factors never develop complex BSI and are over-treated.

In this study, they investigated the safety of a similar 2-week treatment regimen in simple BSI vs. selected groups of BSI with a high risk for complicating spread. They found comparable relapse rates and mortality (both infection-specific and all-cause of 19% vs. 15%). These results suggest that it is safe to withhold preventive measures (prolonged antibiotic regimen) in high-risk patients with negative [^18^F]FDG-PET/CT scans.

Yildiz et al. (17) compared high-risk patients infected with *S. aureus* in a retrospective case–control setup, i.e., 48 cases undergoing [^18^F]FDG-PET/CT versus 54 controls without scans. Overall, mortality was 31%, which was reduced significantly from 44% in controls to 17% in cases, and the reduced mortality remained at 30 days, 90 days, and 1 year. This is in accordance with the most prevalent findings being spondylodiscitis, bone or joint prosthesis infections, vascular graft infections, and abscesses; findings that all require more aggressive treatment such as surgery, removal of prostheses, or prolonged antibiotic regimen to be controlled.

## Impact on treatment modifications and outcome

A small group of studies looked at both treatment modifications and outcomes.

The first study by Ghanem-Zoubi et al. (18) prospectively recruited patients with *S. aureus* BSI for a matched cohort to compare 149 patients who underwent [^18^F]FDG-PET/CT with 149 who did not. [^18^F]FDG-PET/CT found a relatively high number of foci initially not suspected which all required longer antibiotic regimens (e.g., bones) or surgical interventions (e.g., soft tissue abscess drainage). Thus, patients of the intervention group received antibiotics for longer periods (42 days versus 19 days), and 18% underwent focus controlling procedures as a direct consequence of [^18^F]FDG-PET/CT findings. Consequently, the intervention group had significantly lower 90-day mortality (14%) compared with the controls (29%). Interestingly, differences in baseline characteristics were skewed toward potentially more severe disease in the intervention group, e.g., more long-term bacteremia and thus higher rates of high-risk *S. aureus* BSI, which should actually have skewed the mortality toward a more favorable outcome for the control group.

Berrevoets et al. (3) included consecutive patients in a retrospective cohort of in-patients with high-risk *S. aureus* BSI and an [^18^F]FDG-PET/CT (*n* = 99) and compared it with a control group who did not undergo [^18^F]FDG-PET/CT (*n* = 49). In the case group, metastatic infection was present in 73%, most without any diagnostic clues. Again, many foci were located in organs or tissue requiring prolonged antibiotics or intervention (e.g., bones, joints, and abscesses), and 104 treatment modifications were recorded in 74 patients (e.g., escalation/de-escalation of antibiotics or invasive interventions). The overall relapse rates were 2.2% (4/184), whereas the 3-month mortality was 19% (35/184). However, comparing the two groups, *S. aureus*-related 3-month mortality was only 12% in the PET group compared with 33% in the no PET group. In a univariate analysis, the performance of an [^18^F]FDG-PET/CT was one of the factors associated with improved survival.

The same group performed a study in a similar setup but looked at the impact on the outcome of de-escalation treatment regimens in a retrospective cohort of consecutive in-patients with complicated *S. aureus* BSI (19). A dreaded complication of *S. aureus* BSI is infective endocarditis, and according to guidelines, complicated *S. aureus* BSI requires prolonged antibiotics for 4–6 weeks. Some studies suggest a switch from an IV regimen to oral antibiotics after 2 weeks to shorten hospital stay, reduce patient inconvenience, and reduce costs. This study tested the safety of this approach in 106 patients with complicated *S. aureus* BSI without evidence of infective endocarditis or other endovascular foci on echocardiography and [^18^F]FDG-PET/CT. They compared the switch strategy (*n* = 60) with continuous IV treatment (*n* = 46) and found no relapses in either group. Although mortality was lower in the switch group (6.6% vs. 13.3%), results were not statistically significant, but the duration of hospital stay was significantly shorter (17 days vs. 29 days) (19).

It is important to note that continuous or prolonged IV treatment was at the discretion of the treating physician, and the continuous IV treatment group included significantly more high-risk patients. Nonetheless, the IV-to-oral switch of antibiotic regimen is feasible and safe in complex, metastatic high-risk *S. aureus* BSI without signs of endovascular foci on echo and [^18^F]FDG-PET/CT.

## Conclusion/perspectives

[^18^F]FDG-PET/CT has an overall clinical impact on up to half of the patients; more specifically, it leads to changes in treatment in 15–73% of patients, and as a result of these management changes, reduced relapse and mortality of 2–3-fold are observed (Table 2).

**Table 2:** Main conclusions on [^18^F]FDG-PET/CT in bloodstream infections.

Important patient populations	Suspected complex blood stream infections Patients at high risk of metastatic spread Blood stream infections in ICU-patients
Potential impact on patient management, treatment strategy, and patient outcome	Directing the diagnostic process towards more specific diagnostics Modify treatment regimen Guide to invasive treatment (e.g., abscess drainage) Escalation or elongation of therapy (positive findings) De-escalation of therapy (negative scans) Switch from intravenous to oral regimens in limited disease (negative scans in complex bloodstream infections) Decreased mortality when [^18^F]FDG-PET/CT is performed Negative scans may reduce the need for further workup

However, the literature is not without challenges; the studies presented here, although not systematically retrieved, probably comprise the bulk of the clinical studies on the subject and they are relatively small with 18–157 patients. Most are retrospective with only two prospective ones, and the patient populations and the microorganisms covered are highly heterogeneous. Thus, pooling the results and making overall conclusions may be difficult. For instance, two of these studies, albeit the two smallest ones, included ICU patients that are notoriously difficult with a multitude of pitfalls.

A number of knowledge gaps remain. For instance, the timing of [^18^F]FDG-PET/CT — should it be performed upfront or only after a number of futile examinations? Cost issues may suggest the latter to reduce the number of scans, but some proportion of these patients end up in the PET scanner eventually, so some efforts should be put into defining when to use [^18^F]FDG-PET/CT. Moreover, very little is known about the impact of the clinical courses themselves, for instance, the potential impact on diagnostic yield from long-term antibiotics, the duration of symptoms, underlying malignancies, etc.

[^18^F]FDG-PET/CT may save futile exams but may also prompt additional unnecessary exams due to false positive findings. This may affect cost-effectiveness, but although some studies address some of these issues as secondary endpoints, the available data do not allow definite conclusions. More widespread implementation of novel technologies such as digital or whole-body PET scanners may alleviate some of the aforementioned shortcomings, e.g., improved image resolution, reduction of false positive findings, and reduced radiation dose, especially in children, but further studies are needed.

Nonetheless, the available literature leaves no doubt of the potential of [^18^F]FDG-PET/CT in the diagnostic workup of BSI, at least in patients with high-risk complex BSI with risk of metastatic disease, and perhaps especially in *S. aureus* BSI. This was also the conclusion of two very recent systematic and focused reviews compiling much of the same literature (21, 22). [^18^F]FDG-PET/CT reduced relapse rate and mortality due to the detection of additional foci that led to better-guided treatment and ultimately better source control.

## Author contributions

The author confirms being the sole contributor of this work and has approved it for publication.

## Conflict of interest

The author declares that the research was conducted in the absence of any commercial or financial relationships that could be construed as a potential conflict of interest.

## Publisher’s note

All claims expressed in this article are solely those of the authors and do not necessarily represent those of their affiliated organizations, or those of the publisher, the editors and the reviewers. Any product that may be evaluated in this article, or claim that may be made by its manufacturer, is not guaranteed or endorsed by the publisher.

## References

[1] Søgaard M Nørgaard M Pedersen L Sørensen HT Schønheyder HC. Blood culture status and mortality among patients with suspected community-acquired bacteremia: a population-based cohort study. BMC Infect Dis. (2011) 11:139. doi: 10.1186/1471-2334-11-139, PMID: 21599971 PMC3128048

[2] Pijl JP Londema M Kwee TC Nijsten MWN Slart R Dierckx R. FDG-PET/CT in intensive care patients with bloodstream infection. Crit Care. (2021) 25:133. doi: 10.1186/s13054-021-03557-x, PMID: 33827655 PMC8028784

[3] Berrevoets MAH Kouijzer IJE Aarntzen E Janssen MJR De Geus-Oei LF Wertheim HFL. (18)F-FDG PET/CT optimizes treatment in *Staphylococcus aureus* bacteremia and is associated with reduced mortality. J Nucl Med. (2017) 58:1504–10. doi: 10.2967/jnumed.117.191981, PMID: 28336786

[4] Berrevoets MAH Kouijzer IJE Slieker K Aarntzen E Kullberg BJ Oever JT. (18)F-FDG PET/CT-guided treatment duration in patients with high-risk *Staphylococcus aureus* bacteremia: a proof of principle. J Nucl Med. (2019) 60:998–1002. doi: 10.2967/jnumed.118.221929, PMID: 30552202

[5] Vos FJ Bleeker-Rovers CP Sturm PD Krabbe PF van Dijk AP Cuijpers ML. 18F-FDG PET/CT for detection of metastatic infection in gram-positive bacteremia. J Nucl Med. (2010) 51:1234–40. doi: 10.2967/jnumed.109.072371, PMID: 20660384

[6] Muñoz P Cruz AF Rodríguez-Créixems M Bouza E. Gram-negative bloodstream infections. Int J Antimicrob Agents. (2008) 32:S10–4. doi: 10.1016/j.ijantimicag.2008.06.015 18775648

[7] Todorovic Markovic M Pedersen C Gottfredsson M Todorovic Mitic M Gaini S. Focus of infection and microbiological etiology in community-acquired infections in hospitalized adult patients in the Faroe Islands. BMC Infect Dis. (2019) 19:16. doi: 10.1186/s12879-018-3650-3, PMID: 30612543 PMC6322335

[8] Laupland KB Church DL. Population-based epidemiology and microbiology of community-onset bloodstream infections. Clin Microbiol Rev. (2014) 27:647–64. doi: 10.1128/CMR.00002-14, PMID: 25278570 PMC4187633

[9] Saginur R Suh KN. *Staphylococcus aureus* bacteraemia of unknown primary source: where do we stand? Int J Antimicrob Agents. (2008) 32:S21–5. doi: 10.1016/j.ijantimicag.2008.06.008, PMID: 18757183

[10] del Rio A Cervera C Moreno A Moreillon P Miró JM. Patients at risk of complications of *Staphylococcus aureus* bloodstream infection. Clin Infect Dis. (2009) 48:S246–53. doi: 10.1086/598187 19374580

[11] Rubinstein E. *Staphylococcus aureus* bacteraemia with known sources. Int J Antimicrob Agents. (2008) 32:S18–20. doi: 10.1016/j.ijantimicag.2008.06.006 18715760

[12] Kluge S Braune S Nierhaus A Wichmann D Derlin T Mester J. Diagnostic value of positron emission tomography combined with computed tomography for evaluating patients with septic shock of unknown origin. J Crit Care. (2012) 27:316.e1–7. 10.1016/j.jcrc.2011.10.004 22176803

[13] Feld R. Bloodstream infections in cancer patients with febrile neutropenia. Int J Antimicrob Agents. (2008) 32:S30–3. doi: 10.1016/j.ijantimicag.2008.06.017 18778919

[14] Brøndserud MB Pedersen C Rosenvinge FS Hoilund-Carlsen PF Hess S. Clinical value of FDG-PET/CT in bacteremia of unknown origin with catalase-negative gram-positive cocci or *Staphylococcus aureus*. Eur J Nucl Med Mol Imaging. (2019) 46:1351–8. doi: 10.1007/s00259-019-04289-5 30788532

[15] van Leerdam EJ Gompelman M Tuinte RAM Aarntzen E Berrevoets MAH Maat I. Individualizing the use of [(18)F]FDG-PET/CT in patients with complicated *Staphylococcus aureus* bacteremia: experiences from a tertiary care center. Infection. (2022) 50:491–8. doi: 10.1007/s15010-021-01740-4, PMID: 34928493 PMC8942890

[16] Tsai HY Lee MH Wan CH Yang LY Yen TC Tseng JR. C-reactive protein levels can predict positive (18)F-FDG PET/CT findings that lead to management changes in patients with bacteremia. J Microbiol Immunol Infect. (2018) 51:839–46. doi: 10.1016/j.jmii.2018.08.003, PMID: 30190232

[17] Yildiz H Reychler G Rodriguez-Villalobos H Orioli L D'Abadie P Vandeleene B. Mortality in patients with high risk *Staphylococcus aureus* bacteremia undergoing or not PET-CT: a single center experience. J Infect Chemother. (2019) 25:880–885. 31105001 10.1016/j.jiac.2019.04.016

[18] Ghanem-Zoubi N Kagna O Abu-Elhija J Mustafa-Hellou M Qasum M Keidar Z. Integration of FDG-PET/CT in the diagnostic workup for *Staphylococcus aureus* bacteremia: a prospective interventional matched-cohort study. Clin Infect Dis. (2021) 73:e3859–66. doi: 10.1093/cid/ciaa929, PMID: 32639560

[19] Kouijzer IJE van Leerdam EJ Gompelman M Tuinte RAM Aarntzen E Berrevoets MAH. Intravenous to oral switch in complicated *Staphylococcus aureus* bacteremia without endovascular infection: a retrospective single-center cohort study. Clin Infect Dis. (2021) 73:895–8. doi: 10.1093/cid/ciab156, PMID: 33606007 PMC8423458

[20] Berrevoets MAH Kouijzer IJE Slieker K Aarntzen E Kullberg BJ Ten Oever J. F-FDG-PET/CT-guided treatment duration in patients with high-risk *Staphylococcus aureus* bacteremia: a proof of principle. J Nucl Med. (2019) 60:998–1002. 30552202 10.2967/jnumed.118.221929

[21] Buis DTP Sieswerda E Kouijzer IJE Huynh WY Burchell GL Berrevoets MAH. [18F]FDG-PET/CT in *Staphylococcus aureus* bacteremia: a systematic review. BMC Infect Dis. (2022) 22:282. doi: 10.1186/s12879-022-07273-x, PMID: 35331165 PMC8943998

[22] Thottacherry E Cortés-Penfield NW. Evidence of clinical impact supports a new petition for Medicare coverage of 2-[18F]Fluoro-2-Deoxy-D-glucose positron emission tomography/computed tomography in the evaluation of *Staphylococcus aureus* bacteremia: a focused literature review and call to action. Clin Infect Dis. (2022) 75:1457–61. doi: 10.1093/cid/ciac363, PMID: 35535794

